# Efficacy of dexmedetomidine on postoperative pain in patients undergoing gastric and esophageal endoscopic submucosal dissection: a study protocol for a randomized controlled prospective trial

**DOI:** 10.1186/s13063-022-06432-4

**Published:** 2022-06-13

**Authors:** Xin Luo, Li-Xin An, Pei-Shan Chen, Xin-Lu Chang, Yang Li

**Affiliations:** 1 Department of Anesthesiology, Beijing Friendship Hospital, Capital Medical University, No. 95 Yongan Road, Xicheng District, Beijing, 100050 China; 2 Beijing Friendship Hospital, Capital Medical University, Beijing, 100050 China

**Keywords:** Endoscopic submucosal dissection (ESD), Dexmedetomidine, Postoperative pain

## Abstract

**Background:**

Endoscopic submucosal dissection (ESD) is widely used as an effective treatment of early gastric and esophageal tumors, as it is minimally invasive, safe, and convenient. Epigastric pain is a common complication of ESD. In the traditional cognition, the postoperative pain of ESD is not serious and does not attach too much attention. However, previous studies found that the incidence of moderate to severe pain after ESD can be as high as 44.9~62.8%. At present, there is no unified understanding of how to carry out good postoperative analgesia in patients undergoing ESD of stomach and esophagus. The purpose of present study is to investigate the efficacy of intraoperative dexmedetomidine (DEX) using on postoperative pain though observing the postoperative visual analog scale (VAS) score within 48 h after ESD surgery, so as to explore an effective analgesia and anesthetic method in patients undergoing gastric and esophagus ESD.

**Methods/design:**

This study is a prospective, single-center, two-arm, randomized control trail. In total, 120 patients undergoing endoscopic submucosal dissection were stratified by type of surgery (i.e., gastric or esophagus ESD) and randomized into two treatment groups, DEX group (group D, *n* = 60) and control group (group C, *n* = 60). Patients in the experimental group (DEX group) will be administrated a loading dose of DEX at 1 μg/kg for 15 min and a continuous infusion at 0.6 μg/kg/h until 30 min before the end of operation. In control group, the same volume of normal saline was infused. The primary outcome is VAS at 2 h after ESD surgery. The secondary outcome will be VAS at 1 h, 4 h, 6 h,18 h, 24 h, and 48 h, the status of perioperative hemodynamics, the use of remedial analgesics, sedation score, shivering, postoperative nausea and vomiting (PONV), and satisfaction scores of patient and complication of ESD (such as bleeding, perforation, aspiration pneumonia).

**Discussion:**

The results of this study will demonstrate that intraoperative application of DEX is beneficial for postoperative pain treatment in patients undergoing ESD. This study will not only confirm that postoperative pain treatment is necessary for patients undergoing ESD but also provides an effective anesthesia method for postoperative analgesia.

**Trial registration:**

Chinese Clinical Trial Registry, ID: ChiCTR2100043837, registered on March 4, 2021, http://www.chictr.org.cn.

**Supplementary Information:**

The online version contains supplementary material available at 10.1186/s13063-022-06432-4.

## Background

Endoscopic submucosal dissection (ESD) is an effective method for the treatment of early gastric and esophageal tumors, which has more advantages than traditional surgery and endoscopic mucosa resection. ESD can achieves en bloc and complete resection regardless of the lesions size and increases the probability of histologically complete resections [1]. Despite these advantages, ESD is still associated with several complications such as bleeding and postoperative perforation [2]. Besides these major adverse events, some minor complications such as abdominal distension, abdominal pain, stricture, fever, nausea, and vomiting are also encountered commonly after ESD [2, 3]. In the traditional cognition, postoperative pain after ESD is not serious and always be underestimated by clinicians. However, there are still some studies found that the incidence of moderate-to-severe pain after ESD can be as high as 44.9~62.8% and especially in the early postoperative period (within 1–4 h after operation) [4].

Anesthesiologists and endoscopists, considering that these patients who are undergoing ESD often return to the day ward or endoscopic ward after surgery, are worried that the administration of analgesics (such as opioids) will cover up the observation and timely detection of many serious postoperative complications (such as perforation, etc.), so the treatment and intervention of pain is always not active. The occurrence of pain greatly affects the postoperative recovery of patients, reduces patient satisfaction, prolongs discharge time, and increases medical expenses [2–4]. Even, because of the pain, some patients will question the success of the procedure. Because ESD is an organ protection, metachronous lesion is an important issue in monitoring [5]. Even in patients undergoing radical resection, repeated ESD surgery is very common [5]. Patients who have experienced postoperative pain after ESD may fear of subsequent endoscopic procedure or treatment of recurrent lesions. Therefore, effective postoperative pain prevention and management can improve patient satisfaction and compliance with additional treatment or surveillance.

A few studies have reported the incidence of post-ESD pain [4, 6, 7]. However, there is no unified consensus on postoperative analgesia for patients undergoing ESD. According to the research of Lee et al., a single dose of dexamethasone could effectively reduce the postoperative pain after ESD [8]. Kim et al. [9] found that the local use of bupivacaine and triamcinolone acetonide could help to control the abdominal pain after ESD. Even in the latest published “Expert consensus on anesthesia management of common digestive endoscopic surgery” [10], there was not too much description about the pain management after ESD, and it only recommended that nonsteroidal anti-inflammatory drugs (NSAID) can be used for postoperative analgesia. Therefore, how to better prevent and control ESD postoperative pain still need further research.

Dexmedetomidine is a novel selective α2-receptor agonist with sedative, analgesic, and anxiolytic effects, helping reduce the dosage of anesthetics and relatively slightly depresses respiration [11]. Several previous studies have confirmed that perioperative dexmedetomidine infusion has the effect of postoperative analgesia and reducing postoperative VAS [12]. At the same time, a retrospective study also confirmed that dexmedetomidine has a unique advantage in total intravenous anesthesia in gastrointestinal endoscopy [13].

Based on previous studies, our present study’s purpose is to confirm the analgesia effect of intraoperative use of dexmedetomidine on postoperative pain after gastric and esophageal ESD, through observing the postoperative VAS score of patients, to explore the effect of dexmedetomidine on intraoperative anesthesia maintenance and postoperative pain treatment, so as to find a more suitable anesthesia method for patients undergoing ESD surgery.

## Methods/design

This study will be performed at Beijing Friendship Hospital, Capital Medical University, China. The trial will be conducted in accordance with the Declaration of Helsinki together with the CIOMS Principles of the International Guidelines for Biomedical Research Involving Human Subjects. This trial has been approved by the Ethics Committee of Beijing Friendship Hospital, Capital Medical University (Approval No: 2021-P2-003-01), and has been registered in the Clinical Trial Registry (Chictr), registration number: ChiCTR2100043837. The (SPIRIT) 2013 Checklist is listed in Additional file 1.

### Trial design

**Fig. 1:**
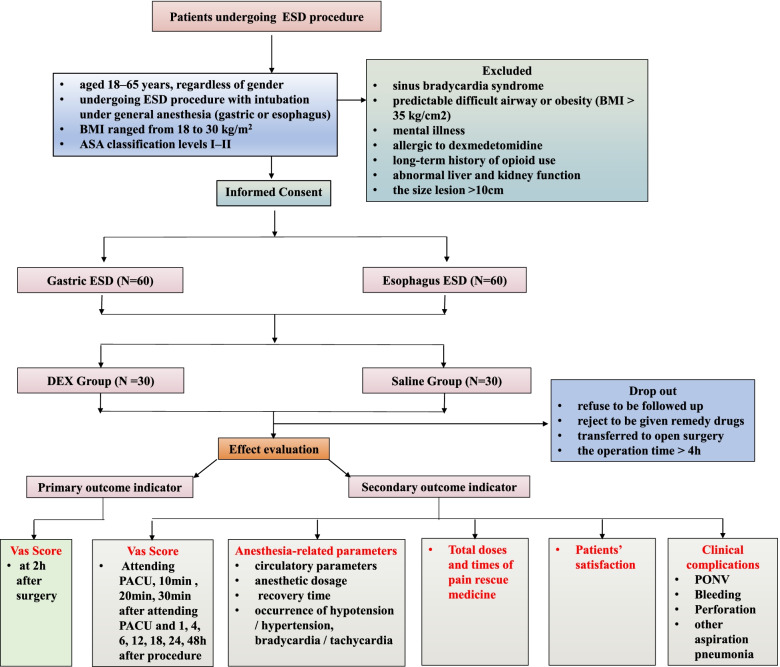
Consolidated Standards of Reporting Trials (CONSORT) diagram of this trial. ESD, endoscopic submucosal dissection; ASA, American Society of Anesthesiologists; BMI, body mass index; DEX, dexmedetomidine; VAS, visual analog scale; PACU, post-anesthesia care unit; PONV, post-operative nausea and vomiting

This prospective, single-center, randomized, controlled, double-blinded trial (Fig. 1) will verify the following hypothesis: perioperative dexmedetomidine infusion will alleviate postoperative pain in patients undergoing gastric or esophageal ESD procedure. Data analysis will be collected according to the superiority principle. The study will sustain for 12 months, and all the selected individuals will be randomly stratified by type of ESD surgery (i.e., gastric or esophageal procedure) assigned into two groups: the dexmedetomidine treatment group (D group) and the saline group (C group). The observatory will conduct screening on basis of the established criteria and pre-standard treatment plan. Data collection will start from the accumulation of basic data until the end of follow-up (Table 1).

**Table 1:**
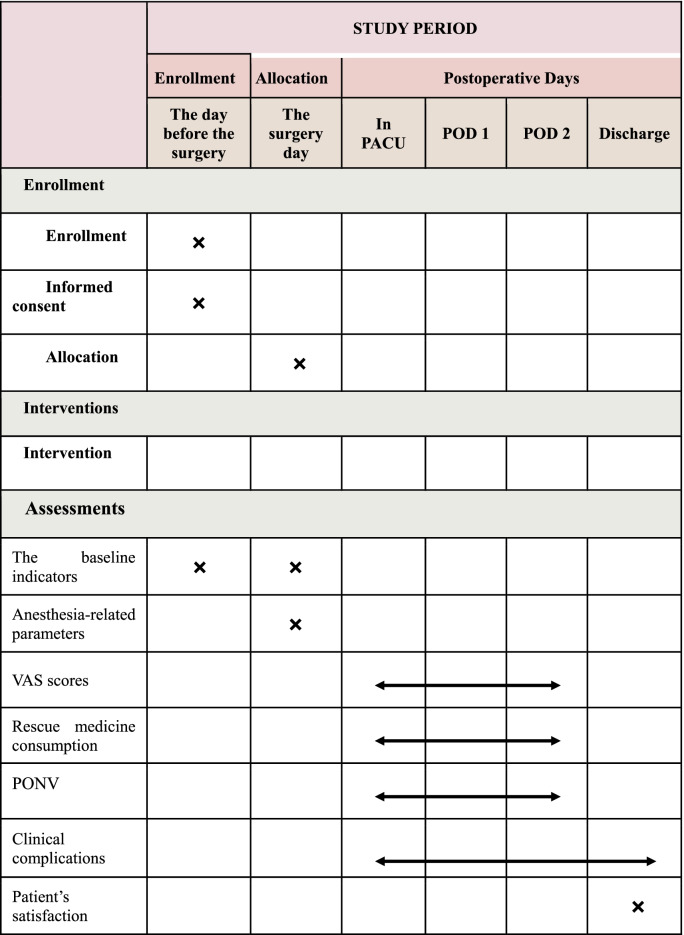
Standard Protocol Items: Recommendations for Interventional Trials (SPIRIT) schedule for enrollment, interventions and assessments

*PACU* post-anesthesia care unit, *POD1* post-operative day 1, *POD2* post-operative day 2, *PONV* post-operative nausea and vomiting

### Randomization and blinding

This is a double-blinded study; participants as well as implementers and observers are also blinded. Randomization will be conducted by a computer-generated blocked randomization sequence with 30 blocks of four patients per block. All participants conforming to the inclusion criteria are randomly assigned to dexmedetomidine group or the saline group at a ratio of 1:1. Allocation will proceed with numbered, sealed, and opaque envelopes. Patient data, anesthesia data, and postoperative recovery data are written in the case report forms (CRF). The blind bottom shall not be known without reason during the trial. Participants will be distributed and assigned in numerical order. The outcome will be assented by assessors, and the data will be calculated by statisticians who will not participate in the treatment; the outcome evaluation and the statistical analysis are announced independently. All original records including informed consent and CRF together with related letters will be reserved for 10 years and then destroyed according to the standards of hospital.

### Study participants and recruitment

We will recruit 120 patients aged 18–65 years, ASA grade I–II scheduled to undergoing gastric or esophageal ESD procedure. These patients will be recruited from Beijing Friendship Hospital of Capital Medical University after they achieve the eligibility criteria and sign the informed consent. We plan to enroll the first patient on March 20, 2021, and finish recruiting on March 31, 2022. All participants will sign the informed consent form as for participating in this clinical trial, collecting and using participant data.

#### Inclusion criteria

Inclusion criteria are as follows: (1) aged 18–65 years, regardless of gender; (2) undergoing ESD procedure with intubation under general anesthesia (gastric or esophagus); (3) body mass index (BMI) ranged from 18 to 30 kg/m^2^ (BMI = weight in kilograms /height in meters squared); (4) American Society of Anesthesiologists (ASA) classification levels I–II; and (5) volunteering to participate in this observation and sign the informed consent.

#### Exclusion criteria

Exclusion criteria are as follows: (1) sinus bradycardia; (2) sick sinus syndrome; (3) predictable difficult airway or obesity (BMI > 35 kg/cm^2^); (4) mental illness; (5) allergic to dexmedetomidine; (6) long-term history of opioid use; (7) abnormal liver and kidney function; and (8) the size lesion > 10 cm.

#### Discharge criteria

Discharge criteria are as follows: (1) requirement for individuals to withdraw during the trail; (2) rejection of application of remedy medication after surgery; (3) violation of the test program; (4) occurrence of serious adverse events (AEs); (5) transferred to open surgery; and (6) the operation time > 4 h.

### Standard procedures

In this study, except for experimental drugs, all anesthesia management (consist of induction and maintenance of anesthesia, recovery period, treatment of postoperative pain and rescue of adverse events) are performed by a relative steady anesthesia team according to treatment routines without any restriction.

The following approaches are suggested:

1. After admission into operation room, patients are routinely monitored blood pressure, electrocardiogram, pulse oxygen saturation, and BIS.
2. Rapid sequence induction will be used as anesthesia induction strategy. Agents for anesthesia induction include midazolam 0.03 mg/kg, remifentanil 1-2 μg/kg, rocuronium 0.6–0.8 mg/kg, and propofol 1–2 mg/kg. After anesthesia agents are given intravenously; then, mechanical ventilation will be performed after tracheal intubation.
3. Anesthesia is maintained with total intravenous anesthesia by infusing propofol, remifentanil, and the experimental drug. Anesthetic depth was titrated to maintain a bispectral index range from 40 to 60 and blood pressure within 20% of baseline during procedure.
4. The compensated tidal volume is set to 6–8 ml/kg (adjusted body weight, ABW) and the respiratory rate to 12–15 breaths/min targeting P_ET_CO_2_ maintenance at 35–45 cmH_2_O.

### Intervention

A nurse not attending the anesthesia of this study open a sealed envelope just before anesthesia and prepare the experimental agent. In the D group, the experimental agent is dexmedetomidine which is provided in a 50-ml syringe (4 μg/ml) and then is infused by the attending anesthesiologist at a bolus rate of 1 μg/kg intravenously over 10 min in induction of anesthesia followed at the infusion rate of 0.6 μg/kg/h during maintenance of anesthesia. Dexmedetomidine administration discontinues at 30 min before the end of procedure. In the control group (C group), the experimental agent changes into saline and is also prepared in a 50-ml syringe. The anesthesiologist will still infuse experimental agent at the same bolus rate and infusion rate with D group. And saline infusion stops before 30 min ending of the procedure.

In this study, no paravertebral block, epidural, TAP, or other nerve block is added during the procedure. A single dose of tramadol (50 mg) is administered intravenously at the moment of the experimental agent discontinuance. A single dose of omeprazole (40 mg) is administered at 2 h after the ESD proceed.

### Postoperative analgesia measures and remedial plan

(1) The postoperative analgesia remedy is as follows: The visual analog scale (VAS) will be recorded after procedure. If VAS score ≥ 4 points or the patients asked for analgesia, morphine 1 mg will be administered. The dose and times will be recorded of rescue medicine. (2) Remedy of nausea and vomiting: the 4-point scale will be used for nausea and vomiting score. The 4-point scale is as follows: 1 = absent, 2 = mild nausea, 3 = severe nausea, and 4 = vomiting. When nausea and vomiting score ≥ 2points, ondansetron 4 mg will be given to participants and repeated if symptoms did not relieve. The remedial application of medicine should be taken notes in the remedial medication list. (3) Concomitant treatment record: concomitant treatment during the trial period (from the start of procedure to 2 days after surgery).

### Outcome assessment

A blind observer from the Data Monitoring Committee (DMC) will gather all outcome data after procedure. The primary outcome of this study is the VAS score at 2 h after surgery. The secondary outcomes include as following: (1) anesthesia-related parameters, circulatory parameters, anesthetic dosage, recovery time, and occurrence of hypotension/hypertension, bradycardia/tachycardia, will be recorded continuously. (2) Total doses and times of pain rescue medicine during 2 days after procedure. (3) Patients’ satisfaction of anesthesia and ESD procedure. (4) Clinical complications: nausea and vomiting, bleeding, perforation, and other aspiration pneumonia. (5) VAS sores at the moment as entering PACU and awake and 1, 4, 6, 12, 18, 24, and 48 h after procedure. All outcomes will be followed up and evaluated by a special medical team.

### Sample size calculation

According to our pre-experimental results, VAS scores at 2 h after surgery in the D group was a significant difference from the control group. We hypothesized that after intraoperative administration of DEX, the VAS score at 2 h in the D group will be significantly lower than that in the C group.

During our pre-experiment, we found that patients undergoing esophageal ESD surgery had significantly more severe pain than patients undergoing gastric ESD surgery. The degree of pain was closely related to the operation type. Therefore, in order to avoid the interference caused by the type of surgery, we decided to calculate the sample size of patients with gastric ESD and esophageal ESD respectively.

In our pre-experiment, the VAS score at 2 h after surgery in patients undergoing gastric ESD in DEX groups vs control group was 0.67 ± 1.63 vs 2.38 ± 1.06. We used the PASS 20.0 software to estimate the sample size. With a probability of *α* = 0.05, *β* = 0.1, and power 0.90, the sample size was 15.

And the mean ± standard of VAS score at 2 h after surgery in the DEX group and control group in patients undergoing esophageal ESD was 2.83 ± 0.57 vs 3.70 ± 1.20. When *α* = 0.05 and *β* = 0.1, 26 cases are needed in each group in patients undergoing esophageal ESD procedure by PASS 20.0.

Considering the ratio between each group by 1:1, we choose the bigger sample size 26 as the sample size in each group; moreover, considering 15% patient loss, 30 cases were required in each group. Eventually, there are 60 cases in the DEX group (30 in gastric ESD vs 30 in esophageal ESD procedure) and 60 cases in the control group. There are 300–400 cases of upper gastrointestinal ESD procedure in our digestive endoscopy center per year, so the trial period is set 12 months consequently.

### Statistical methods

Statisticians negotiating with the research team establish databases and analyze statistical data, and the SPSS 20.0 statistical software was used for conducting statistics. The most of the source data will be registered onto CRF, and the pattern of missing data will be evaluated before data analyzing. All *P*-values were unilateral, and *P* < 0.05 was considered significant. The outcomes which are normal distribution data will be expressed as the mean ± standard deviation. Statisticians used *t* test for analysis of normal distribution data. Mann-Whitney *U* test and Wilcoxon signed rank test will be used for analysis of skew data. Fisher’s exact test will probably be the analysis method for non-normal distribution data. Subgroup analysis will be conducted according to different procedure type.

### Data collection methods and monitoring

The DMC is consisting by a doctor whose major is anesthesia in responsible for data collection and sorting, a scientific researcher, and a statistician. The statistician make consultation with the main researchers, generate the allocation sequence, establish the database, and analyze the statistics with application of SPSS statistical analysis system. The anesthesiologist from the DMC will be responsible for participant enrolled, obtaining informed consent, recording data of the participant, the actual number of individuals enrolled, the exclusion cases, basic characteristics, incidence of complications and related treatment, and comprehensive efficacy evaluation. The demographic characteristics, medical history, and treatment history of the patients will be recorded. In the middle of the trial, that is when 30 gastric ESD and 30 esophageal ESD are enrolled, the research team will conduct unblinding and interim analysis. If the analysis result after unblinding is consistent with the hypothesis, the test will be continued. If it is inconsistent or even contrary, the expert committee will be consulted to decide whether to continue the test, expand the sample size, or terminate the test. The protocol non-adherence and missing data will be discharged, and the data will be deleted according to the standards of hospital. Any protocol modification will be informed to IRB, trial registries through email, and inform the participants directly. At the end of the study, the original data and results will be reserved by the scientific research management committee; they will be private data before the results are published.

### Safety evaluation

In the process of clinical research, researchers should write the AE record form truthfully and in detail, including the clinical features, occurrence time, severity, duration, related treatment, and outcomes of AEs. When an AE occurs, the observing clinician must decide whether to withdraw the participants from the observation or not according to the condition. All AEs should be treated and documented in detail until the individual’s symptom is properly resolved or the individual recover to a stable condition; if laboratory tests are seriously abnormal, they should be treated achieving pretreatment level in time.

## Discussion

This study can fully test the hypothesis that intraoperative administration of dexmedetomidine may improve the treatment of postoperative pain in terms of VAS score, the maintenance of anesthesia in patients undergoing gastric and esophagus ESD.

Endoscopic submucosal dissection (ESD) has become the main treatment practice for early-stage gastric and esophagus neoplastic lesions since it was developed in the late 1990s in Japan. In the beginning, it was related to an unusual endoscopic technique and had a higher risk for complications such as bleeding and perforation [2]. With attaching more and more attention, the success rate and safety of ESD have currently improved to favorable levels. As its great merit, ESD became a standard treatment in Japan and other East Asian countries [2]. Facciorusso et al. [14] reported that ESD was entirely effective for early gastric cancer with higher en bloc and had histologically complete resection rate and lower local recurrence. In another meta-analysis about the treatment of superficial esophageal cancer, Guo et al. [15] found that ESD was associated with high rate of en bloc resection (97.1%) and curative resection rate (92.3%) for superficial esophageal cancer. Compared to conscious sedation, general anesthesia with endotracheal intubation by anesthesiologist can offer optimal visualization with minimal patient movement [16, 17]. Moreover, there is little risk of aspiration pneumonia with intubation during the ESD procedure, and the operator can focus on ESD maneuvers without attending to management of anesthesia with assistant of an anesthesiologist. Therefore, in this trial, ESD procedure was performed under general anesthesia with endotracheal intubation which can effectively maintain optimal visualization and prevent complication such as aspiration pneumonia.

With the development of endoscopic equipment and clinical operative experiences of endoscopists, the incidence of severe post-ESD complications such as hemorrhage and perforation has steadily declined [18]. However, there are still many patients complained about abdominal pain after ESD procedure and physicians tend to neglect post-ESD pain. Jung et al. found that the incidence of moderate to severe pain induced by ESD requiring painkillers was 53.8% [19]_;_ female sex, distal stomach tumors, baseline dyspeptic symptoms, and positive acid infusion were factors that correlated with medium-to-severe pain after ESD [7, 19]. The same conclusion was obtained in Kim et al.’s study [4]. The rate of post-ESD pain is high enough for physician to pay more attention and make active pain management with effect. Understanding the influencing factors of pos-ESD pain and active treatment can make patients obtain good surgical experience and reduce fear. Pain after ESD may be due to residual mucosal defects (i.e., edema and inflammatory artificial ulcers), exposure to gastric acid, osmotic or chemical effects of submucosal fluid injection, or burn injury during ESD. Low visceral pain threshold to acid, or so-called acid allergy, may also be related to the development of epigastric pain after ESD [4, 7, 19].

Based on these mechanisms, some researchers investigated valid measures to alleviate post-ESD pain. Jung et al. [19] also found that intravenous proton pump inhibitor (PPI) prevented acid hypersensitivity and resulted in a lower frequency of ESD-related pain. Some investigators tried to manage ESD-related pain by utilization of local lidocaine injection or by application of a transdermal fentanyl patch [6, 20]. Lee et al. [8] tried to use single-dose postoperative intravenous dexamethasone relieving pain after ESD procedure which being proved effective. However, at present, there is no unified measure of how to carry out good postoperative analgesia for patients undergoing ESD operation of stomach and esophagus. Even in the latest expert consensus on anesthesia management of common digestive endoscopic surgery [10], only recommended non-steroidal anti-inflammatory drugs (NSAIDS) to control post-ESD pain.

ESD is a minimally invasive treatment which owns characteristics of fast postoperative recovery and less hospital stay compared with traditional surgery. In consideration that most of the ESD procedure are performed with intubation under general anesthesia, clinicians should not only concern the postoperative complications of ESD but also adverse events of general anesthesia such as hypoxemia, respiratory depression, delayed recovery, agitation, and regurgitation aspiration [21]. As a consequence, acetaminophen, NSAIDS, and other kinds of medicine owning moderate analgesia effects become the optional choice in ambulatory surgeries such as endoscopic treatment rather than opioids which should be avoided or reduced for analgesia [22]. Dexmedetomidine exerts special analgesia effect reducing anxiety and opioid-sparing effect [23]. When compared with an opioid alone, application of dexmedetomidine lead to lower post-operative pain intensity scores, lower morphine-equivalent consumption, and more patient satisfaction in patient-controlled analgesia. Opioid-sparing effect of dexmedetomidine might be profitable for patients at risk for post-operative respiratory depression [24]. Yoshio et al. [25] found that the combination of DEX and midazolam could provide effective sedation for ESD for esophageal squamous cell carcinoma. Ishibashi et al. [26] similarly showed that DEX used in intubation general anesthesia for ESD was also safe and effective.

In conclusion, considering that there are few reports on postoperative analgesia with dexmedetomidine used in ESD, our aim is to confirm that intraoperative dexmedetomidine can indeed reduce the dosage of opioids and postoperative pain intensity score without adverse reactions. This study will provide further evidence for whether dexmedetomidine should be included in perioperative analgesia agent of ESD, and ultimately as a feasible measure contributed to multi-model analgesia strategy.

### Trial status

The first participant will be enrolled on March 22, 2021, and the first version was developed on January 1, 2021; the protocol version is the first version, and the No is V1.0/2020.12.18. The recruitment will be completed on March 22, 2022. At present, no cases have been formally included, and the trial is in the process of preparation and coordination.

## Supplementary Information

**Additional file 1.**

## Data Availability

After the study is completed and the results is published, the data will be open to the public through email to the research team.

## Abbreviations

ESD: Endoscopic submucosal dissection
DEX: Dexmedetomidine
VAS: Visual analog scale
PONV: Postoperative nausea and vomiting
CRF: Case report forms
BMI: Body mass index
ASA: American Society of Anesthesiologists
CONSORT: Consolidated Standards of Reporting Trials
AEs: Adverse events
ABW: Adjusted body weight
SPIRIT: Standard protocol items: Recommendations for interventional trials
PACU: Post-anesthesia care unit
POD1: Post-operative day 1
POD2: Post-operative day 2
DMC: Data Monitoring Committee
NSAIDs: Non-steroidal anti-inflammatory drugs
